# De Novo Transcriptome Assembly, Functional Annotation, and Transcriptome Dynamics Analyses Reveal Stress Tolerance Genes in Mangrove Tree (*Bruguiera gymnorhiza*)

**DOI:** 10.3390/ijms22189874

**Published:** 2021-09-13

**Authors:** Matin Miryeganeh, Hidetoshi Saze

**Affiliations:** Plant Epigenetics Unit, Okinawa Institute of Science and Technology Graduate University, 1919-1 Tancha, Onna-son 904-0412, Okinawa, Japan; hidetoshi.saze@oist.jp

**Keywords:** mangrove, de novo assembly, transcriptome, RNA-Seq, abiotic stress, salt stress, gene expression

## Abstract

Their high adaptability to difficult coastal conditions makes mangrove trees a valuable resource and an interesting model system for understanding the molecular mechanisms underlying stress tolerance and adaptation of plants to the stressful environmental conditions. In this study, we used RNA sequencing (RNA-Seq) for de novo assembling and characterizing the *Bruguiera gymnorhiza* (L.) Lamk leaf transcriptome. *B. gymnorhiza* is one of the most widely distributed mangrove species from the biggest family of mangroves; Rhizophoraceae. The de novo assembly was followed by functional annotations and identification of individual transcripts and gene families that are involved in abiotic stress response. We then compared the genome-wide expression profiles between two populations of *B. gymnorhiza*, growing under different levels of stress, in their natural habitats. One population living in high salinity environment, in the shore of the Pacific Ocean- Japan, and the other population living about one kilometre farther from the ocean, and next to the estuary of a river; in less saline and more brackish condition. Many genes involved in response to salt and osmotic stress, showed elevated expression levels in trees growing next to the ocean in high salinity condition. Validation of these genes may contribute to future salt-resistance research in mangroves and other woody plants. Furthermore, the sequences and transcriptome data provided in this study are valuable scientific resources for future comparative transcriptome research in plants growing under stressful conditions.

## 1. Introduction

Throughout their evolutionary history, plants have adapted to widely varied and sometimes very stressful environments. The efficiency of their adaptation to environmental stresses (such as extreme temperature, high salinity, and hypoxia), not only depends on the presence of stress response genes, but it also relies on how the expression of those genes is controlled [1]. When plants encounter environmental pressure, the expression of related genes will change accordingly, in order to help plants to overcome and survive the stress, which can especially be important for long living plants that have to cope with different kind of undesirable conditions during their long lifespan [2,3,4]. Salinity is one of the top environmental pressure for plants [5]. Compare to over 10,000 tree species that live at the interface of “non-saline” water and dry land, there are only 80 tree species that have succeeded and survive living in the intertidal “saline” zone. These resilient trees are called mangroves. Mangroves are exposed to many different stress factors such as high salinity, intense UV and anaerobic soils, and have developed some special characteristics such as vivipary and aerial roots in course of their evolution, to adapt to the harsh environment [6,7]. Despite their difficult living conditions, mangroves still manage to provide many ecological services for intertidal life forms, including carbon dioxide removal, sediment accretion, coastal protection and ecosystem productivity [8]. With the forecasted climate change and rising sea level, there is an urgent need to study mangroves as one of the most valuable tropical coast species, in order to make plans to protect these endangered trees [9]. In addition, as salt-tolerant woody plants that deal with daily fluctuation of salinity level in intertidal zone, mangroves make a good model system for studying the dynamics of gene expression under different level of salt stress, and can be studied to identify potential salt stress response genes [10].

Research on mangroves is rare, and the molecular mechanisms behind their tolerance to high salinity environment is still unclear, mainly because of the lack of genome references for these species. De novo RNA-Seq assembly makes the study of transcriptomes for non-model plant species feasible, without availability of reference genomes by enabling an extensive search of their transcriptomes and identifying almost all expressed genes in a plant tissue [11]. Large-scale gene expression analysis may help with understanding the genetic mechanisms of stress responses in plants [12]. It provides a new avenue for study the story of their adaptation on a molecular basis. Research in different plant species have already emphasized the importance of gene expression changes in response to salinity stress (e.g., [13,14,15]).

**Figure 1:**
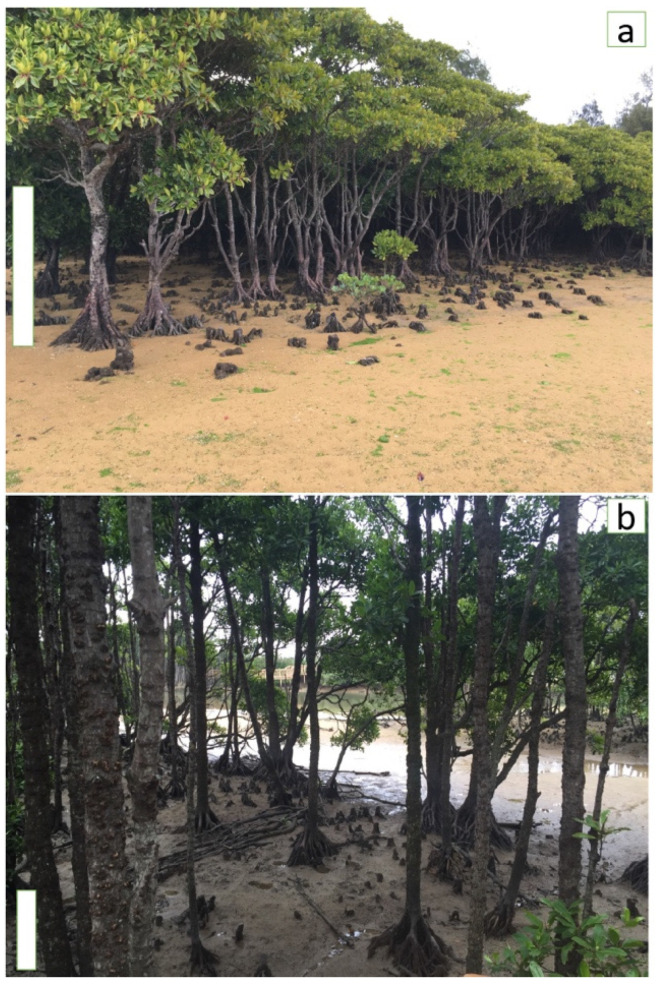
*B. gymnorhiza* trees in the oceanside population (**a**), and the riverside population (**b**). Bars represent about one meter. Oceanside trees appear shorter (1–1.5 m) and the riverside trees grow to be tall at about 7–8 m.

Mangrove communities are known to present gradual phenotypic changes in their structure, such as reduction in biomass and tree height, due to increasing stress factors (e.g., high salinity, strong wind and poor nutrient conditions along the tidal gradients) [16]. Lira-Medeiros et al. [17], have reported a correlation between morphological traits of natural population of a white mangrove tree (*Laguncularia racemosa*), and DNA methylation, where trees growing in contrasting habitats showed striking differences in their morphology which was associated with DNA methylation patterns. In addition, living in intertidal zone, mangroves are naturally subjected to daily variations of water salinity due to sea level oscillations. Therefore, they need to tolerate a wide range of environmental conditions that causes divergent morphological traits depending on their growing environment [18]. In areas with brackish water, and enough nutrients, trees usually fully develop, and some mangrove species even grow to be over 40 m tall. In contrast, in habitats with high saline soil, less nutrients, and periodic drought, they show abnormal development and grow to be of only 1 to 2 m tall, with a shrub-like phenotype [18]. In our study, we used this characteristic to investigate the role of gene expression in adaptation of these trees. We performed a de novo assembly, and annotation of transcriptome, as well as gene expression analyses of two populations of mangrove species, *B. gymnorhiza*, which is one of the widest distributed mangrove trees from the most mangrove-rich family (Rhizophoraceae). The two populations are located in two nearby naturally contrasting habitats, where they are subjected to different environmental stresses such as salinity variation which has caused them to be distinguished by their significant phenotypic differences (i.e., tree height, tree diameter and leaf width) (Figure 1, Table S1). One population (hereafter “riverside”) is close to the mouth of a river and farther from the ocean with the trees growing mostly in brackish water and the other population (hereafter “oceanside”) was located about one km apart and farther from the river in the shore of Pacific Ocean Japan in saline environment. As shown in Figure 1 and Table S1, the individuals are morphologically distinct, having a treelike structure in the riverside and a shrublike phenotype in the oceanside. We aimed to reveal differentiation of gene expression among individuals from the two habitats, at a riverside and ocean side, and investigate if the variation in the expression of stress-response genes is associated with the observed morphological variation of these trees, influenced by their environmental condition.

We specifically asked the following questions: (1) how do mangroves adapt to high-salinity sea water and what are the potential underlying molecular mechanisms? (2) Can transcriptome variation exhibit in some salinity levels but not in others, and can they correlate with phenotypic variation of trees and their level of stress? We identified homologs of genes encoding for salt stress and osmotic stress, which led to the hypothesis of the conserved functions of these genes, which may be used in genetic improvement of plants growing under stressful condition. Validation of these salt-tolerance genes may contribute to future salt-tolerance research in other trees. This study may be a starting platform for deciphering the molecular mechanism underlying the high salinity stress response in mangrove trees. The results may contribute to a better understanding of the adaptive responses in plant species in natural fluctuating environmental conditions.

## 2. Results

### 2.1. Plant Material, Soil Salinity and Morphological Measurements

Newly opened young leaves from the nine mature individuals of *B. gymnorhiza* (*Rhizophoraceae*) trees (five biological replicates from the oceanside and four from the riverside), in the mangrove forest of our study site were collected, frozen in liquid nitrogen, and stored at −80 °C for RNA extraction. The riverside trees were located along the estuary of the river, and the oceanside trees grew along the coastal area of the Japanese Pacific Ocean (see “Materials and Methods” for details about the location). The sampling was performed from 12:30–13:00 on 28 Jun 2019 on a sunny day (temperature: 31 °C—Humidity level 92%, precipitation: 0%) from both oceanside and riverside plants.

The salinity of soil water surrounding the roots was measured using a Refractometer W/ATC 300011, SPER Scientific (Scottsdale, AZ, USA) and was recorded to be 3.5% at the oceanside and 2.0% at the riverside, on the day of sampling. In addition, the salinity of water surrounding the trees in the field, at both oceanside and riverside was measured every fortnight for one year between 12:30–13:00 pm. The average salinity level at the oceanside was 3.5% (35 part per thousands (ppt)), ranging from 3.0% to 4.0%, and at the riverside it was measured to be 2%, ranging from 1.5% to 2.5% (15–20 ppt) (Table S1).

Morphological traits of 30 randomly-chosen *B. gymnorhiza* individual trees from both oceanside and riverside (15 from the oceanside and 15 from the riverside) were measured and recorded at the same day as sampling day. We measured tree height, and tree diameter at breast height (DBH). We also collected 25 leaves from the distal end of lower branches of those 30 trees, in order to measure leaf length, and leaf width. Mean height of the trees appeared to be 1.2 m at the oceanside, and 7.3 m, for the riverside tress (Table S1). Mean tree diameter at breast height, was 95.2 cm for the oceanside and 23.8 cm for the riverside ones. Mean leaf length was measured to be 8.8 cm for the oceanside and 3.7 cm for the riverside trees. Mean leaf width was measured to be 4.5 cm for oceanside and 3.2 cm for the riverside trees. The results were tested for significant differences between sites using the Welch Two Sample *t*-test in R software (available online: http://www.R-project.org—version 3.5.0—April 2018) (Table S1).

### 2.2. High Throughput Sequencing Output and De Novo Transcriptome Assembly

To study the gene expression differentiation between two populations of *B. gymnorhiza* mangrove trees growing in different levels of stress in their natural environment (oceanside vs. riverside), a de-novo transcriptome assembly of the species was generated. Leaves from the nine *B. gymnorhiza* individual trees (five from the oceanside and four from the riverside), were used and generated a total of 199.30 megabase pairs (Mbp) of paired-end raw reads 150 bp long (Table S2). After filtration for removing adaptors, low quality sequences, and short sequences, 180 Mbp of clean reads were obtained. More than 99% of bases in clean reads had a Q value  ≥  20 and more than 97% had a Q value  ≥  30. The GC contents were 43–45% for both raw reads and clean reads (Tables S2 and S3). Initial assembly analysis using Trinity pipeline (version 2.4) (with a minimum contig length of 200 bp and a minimum kmer covariance of 2) [19], generated 254,892 basepair (bp) of total transcripts with an average length of 999.8 bp and a N50 of 1613 bp. After removing transcripts that were shorter than 300 bp, the assembly consisted of 218,920 bp of total transcripts with an average length of 1120 bp and a N50 of 1680 bp. Finally, after clustering redundant transcripts together using CD-HIT program [20], the final assembly contained of 132,268 bp total transcripts with an average length of 1150 bp and N50 of 1786 bp (Table S4). The assembled sequence lengths ranged from the 300 bp cut-off value to a maximum transcript length of 14,969 bp. The majority of the assembled sequences were in the ranges of 300–500 bp, and 501–1000 bp, while the frequency of longer transcripts gradually decreased, and only a minor proportion reached the lengths of above 5000 bp (Figure S1).

### 2.3. Quality Assessment of the Transcriptome Assembly

To evaluate the consistency of the assembly, the filtered unique reads were mapped back to the final assembled transcriptome using the alignment software Bowtie2 (available online: https://sourceforge.net/projects/bowtie-bio/) [21]. The overall alignment rate was >95.46%. About 38.56% of the reads uniquely mapped to a single assembled transcript, whereas 56.90% of the reads aligned to more than one transcript (Table S5). The completeness of the assembled transcripts in terms of expected genes was also checked through the Benchmarking Universal Single-Copy Orthologs (BUSCO) algorithm by comparing the transcripts to the set of Embryophyta genes using BUSCO quality assessment tool [22]. As shown in Table S6, among the 1440 orthologous gene sets of Embryophyta searched, 91.9% (1324 BUSCOs) were “complete” BUSCO copies (i.e., 710 single-copy and 614 duplicated), 3.5% (51 BUSCOs) were “fragmented”, and the remaining 4.6% (65 BUSCOs) were “missing”. These results indicate that the transcriptome is properly assembled and is of good quality. We further searhced for organism distribution in our transcriptome based on BLASTx and BLASTp, and the results assigned *Arabidopsis thaliana* as the species that contained the most homologous genes with *B. gymnorhiza* which is consistent with the previous report for *B. gymnorhiza* [23] (Figure S2, Tables S7 and S8).

### 2.4. Functional Annotation of the Transcriptome by Homology and Gene Ontology (GO)

To determine putative functions of the identified transcripts of *B. gymnorhiza*, and to detect homology between the predicted open reading frames (ORFs)/potential proteins, and the sequences deposited in the universal databases, we adopted the Trinotate pipeline (available online: https://github.com/Trinotate/Trinotate.github.io). ORFs and potential coding sequences were first predicted using the TransDecoder software. From the initial leaf unitranscript catalogue of 132,268 non redundant (NR) unique sequences, 109,506 ORFs, and 76,565 potential proteins were predicted. The retrieved nucleotide sequences and putative protein sequences were then functionally annotated using the Trinotate pipeline, searching for nucleotide (BLASTx) and protein (BLASTp) homology (e-value < 1 × 10^−5^) against the UniProtKB/Swiss-Prot, and UniRef90 databases. In addition, the presence of known functional protein domains and potential signal peptides were analysed using the Pfam protein domain database and SignalP, respectively. In total, 94,455 nucleotide sequences out of the 132,268 transcripts (71.41%) and 62,285 protein sequences out of the 109,506 ORFs (56.87%) displayed significant homology when aligned against the UniProtKB/Swiss-Prot database through BLASTx and BLASTp searches, respectively. 40,303 (36.80%) unique Pfam protein motifs could be assigned, and 2900 (2.64%) protein sequences were predicted to have signal peptides (Table S9). Among the Pfam domains, the most abundant classes were (ResIII): Type III restriction enzyme: SNF2 family N-terminal domain, Transferase family, and (LigB): Catalytic LigB subunit of aromatic ring-opening dioxygenase, Anaphase-promoting complex subunit, CCCH Zinc finger proteins. These protein domains can be found in proteins involved in various functions such as transcription regulation, dioxygenase activity, protein degradation, and mRNA binding. Details regarding the annotation of the transcriptome, including Swiss-Prot hits, GO, KEGG and eggNOG mappings can be found in the Table S10. PFAM predicted protein domains, as well as information regarding signal peptides and transmembrane domains are also shown in the Table S10. Taken together, we have assembled a good quality transcriptome for the mangrove tree *B. gymnorhiza* and comprehensively annotated the transcripts using well-established Trinotate pipelines.

**Figure 2:**
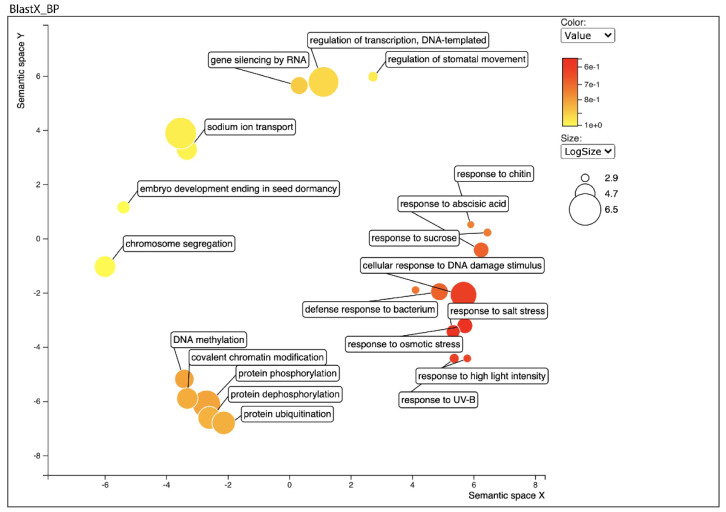
Biological process category of Gene Ontology (GO) enrichment analysis of leaf transcriptome BLAST Top-Hits species distribution when compared with nr database using REVIGO. Circles in closer proximity have more closely related GO terms. The size of the circles indicates the number of GO terms. The colour of the circle represents the significance of the enriched GO terms.

Furthermore, to further functionally characterize the *B. gymnorhiza* leaf transcriptome, we related the detected transcripts to GO and biological pathways using the DAVID Gene Ontology [24], and the REVIGO [25] software. First the unitranscripts and predicted proteins were assigned to genes by BLASTx and BLASTp searches against the UniProtKB/Swiss-Prot database (Table S11 and Table S12, respectively). GO analysis of BLASTX data, revealed 51,066 sequences associated to 829 GO terms. Among the three main categories, the Biological Process (BP) category was the most abundant (16,242 sequences, 435 GOs), followed by Molecular Function (MF) (12,679 sequences, 208 GOs) and Cellular Component (CC) (22,145 sequences, 186 GOs) categories (Tables S13–S15). GO analysis of BLASTP output revealed 44,446 sequences associated with 829 GO terms. Among the three main categories, BP category was the most abundant (13,883 sequences, 405 GOs), followed by (MF: 11,243 sequences, 189 GOs) and (CC: 19,320 sequences, 176 GOs) categories (Tables S16–S18). Within the BP category, regulation of transcription (24.11%), protein phosphorylation and protein ubiquitination (8.14%), and response to salt stress and abscisic acid (5.48%), were the most represented. In addition, among all BP categories 2207 (27.45%) of them were in the category related to response to stress and stimulus. Likewise, genes encoding ATP binding, protein binding, DNA binding, ion binding and mRNA binding (81.61%) and genes encoding proteins related to catalytic activities (16.41%) were the most abundant in the MF category. In the CC category, nucleus (39.70%), plasma membrane (19.92%), cytoplasm (19.75%), and chloroplast (18.61%) were the most abundantly represented GO terms (Figures S3 and S4). Particularly, proteins with stress response activities were highly abundant in the transcripts catalogue (Figure S3 and Figure 2).

Next, to identify the functional biological pathways in *B. gymnorhiza*, the assembled transcripts were mapped against the Kyoto Encyclopedia of Genes and Genomes (KEGG) database. The 109,506 unigenes were divided into 30 major metabolic pathways. Among the 30 KEGG pathways analyzed (Tables S19 and S20), metabolic pathways, biosynthesis of antibiotics, followed by carbon metabolism, plant-pathogen interaction, endocytosis, ubiquitin mediated proteolysis, amino sugar and nucleotide sugar metabolism and mRNA surveillance pathway (1976 sequences), were the most represented pathways. Furthermore, glycerophospholipid metabolism (66 sequences), peroxisome (46 sequences), glycine, serine, and threonine metabolism (49 sequences), glycerolipid metabolism (43 sequences), were among the most represented (Figure S5).

### 2.5. Differential Expression Analysis and Functional Classification

**Figure 3:**
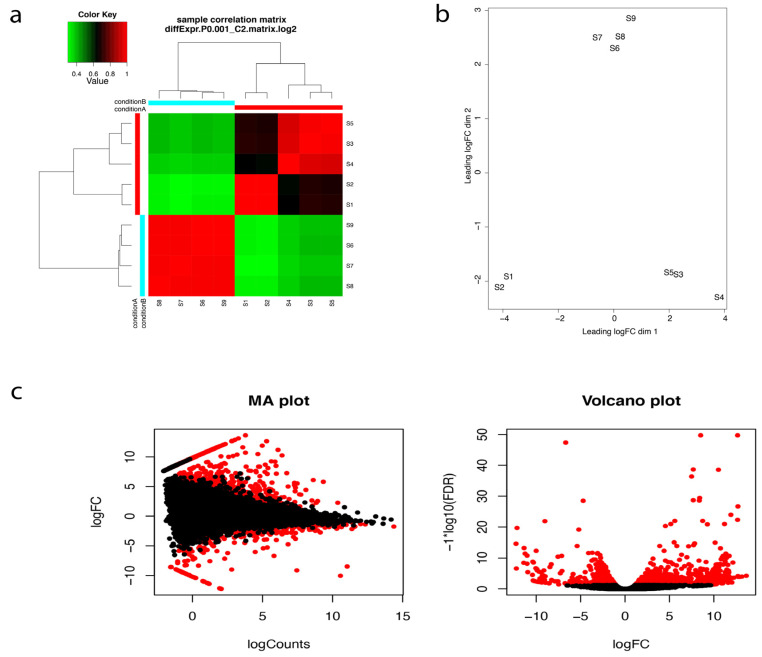
(**a**) Correlation matrix showing relationship between all samples as well as replicates. S1–S5 are the five replicates from the oceanside and S6–S9 are the four replicates from the riverside. Condition A and Condition B refer to the Oceanside and the Riverside respectively (**b**) Principal component analysis and (**c**) Pairwise comparisons of transcript abundance. MA plots showing average log fold change (logFC) vs. average log of counts among oceanside vs. riverside transcripts, across replicates. Volcano plots showing differentially expressed transcripts in relation to FDR (False discovery rate) for oceanside vs. riverside transcripts. Features found DE at FDR < 0.05 are coloured red. Features with *p*-values at most 1 × 10^−3^ and at least 2^2^-fold change are differentially expressed.

After transcriptome assembly and annotation, differential expression analyses were performed. The program RNA-Seq by Expectation-Maximization (RSEM) (available online: http://deweylab.github.io/RSEM/package) [26], was used for transcript abundance quantification. We then checked for the correlation between the replicates for all the RNA-seq samples using Trinotate built in script (Figure 3a). PCA analysis and correlation matrix showed a good correlation between the replicate sets for each of the nine samples (Figure 3b). Empirical Analysis of Digital Gene Expression (EdgeR; available online:http://biocon-ductor.org/packages/release/bioc/html/edgeR.html) R package [27], was used for differential expression analyses and differentially expressed transcripts (DETs) at a minimum fold change of 2^2^ with *p*-values at most 1 × 10^−3^ were extracted. 464 differentially expressed genes were identified (Table S21, Figure 3c).

**Figure 4:**
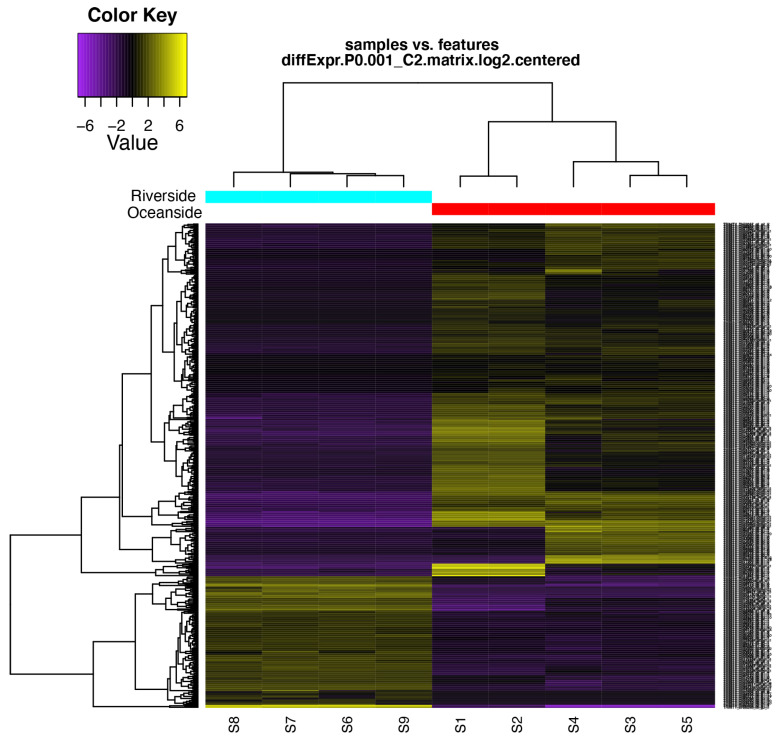
Hierarchical clustering of differentially expressed transcripts and oceanside-riverside *B. gymnorhiza* leaf samples. Heatmap showing the relative expression levels of each transcript (rows) in each sample (columns). Rows and columns are hierarchically clustered. Expression values (FPKM) are log_2_ –transformed and then median-cantered by transcript. S1–S5 are the five replicates from the oceanside and S6–S9 are the four replicates from the riverside.

Among them, 387 genes were upregulated (Table S22) and 116 downregulated (Table S23) in oceanside plants. To further elucidate the functions of the differentially expressed transcripts, GO enrichment analysis was performed and DETs were annotated according to biological processes, cellular components, and molecular functions (Table S24). The GO terms of the differential expressed transcripts obtained from the EdgeR algorithm are presented in (Figures S6–S8) which reflect the effect of stresses (e.g., high salinity) according to the distance from the ocean. The DE features were partitioned into clusters with similar expression patterns (Figure 4 and Figure S9). For upregulated transcripts, GO analysis revealed 4244 sequences associated to 386 GO terms. Among the three main categories, BP category was the most abundant (1815 sequences, 211 GOs), followed by MF (1198 sequences, 142 GOs) and CC (587 sequences, 25 GOs) categories (Tables S25–S27). Likewise, for the downregulated transcripts, GO analysis revealed 2818 sequences associated to 115 GO terms. Among the three main categories, CC category was the most abundant (1528 sequences, 32 GOs), followed by BP (929 sequences, 61 GOs) and MF (347 sequences, 16 GOs) categories (Tables S28–S30, Figures S6–S8).

Among upregulated transcripts: within the BP category, biological process, developmental process, small molecule metabolic process, and catabolic process were most represented, while many BP categories of responses to stimulus, such as response to salt stress, response to osmotic stress were also represented. Interestingly, a negative regulation of DNA methylation was observed which may imply the role of DNA methylation in coping with stressful conditions (Figure S7). In the CC category, membrane part, integral component of membrane, extracellular region, bounding membrane of organelle, and cell wall were the most abundantly represented GO terms (Figure S7). Likewise, genes encoding catalytic activity, oxidoreductase activity and genes encoding proteins related to transporter activity were most abundant in the MF category, along with less abundant and yet significant representatives such as RNA polymerase II transcription factor activity, sequence-specific DNA binding, transcription factor activity, and protein binding (Figure S7). Among downregulated transcripts: within the BP category, biological process, metabolic processes, macromolecule modification, and protein modification process, were most represented, while regulation of response to stimulus categories, RNA modification, ubiquitin-dependent protein catabolic process, and methylation were also represented (Figure S8). In the CC category, cell part, intracellular part, and organelle were the most abundantly represented GO terms (Figure S8). Genes encoding nucleic acid binding, protein binding and RNA binding were most abundant in the MF category, along with less abundant and yet significant representatives such as methyltransferase activity, and UDP-glycosyltransferase activity (Figure S8). These numbers indicate the importance of essential metabolic and biosynthetic processes in *B. gymnorhiza*. DETs were also annotated into 46 KEGG pathways. KEGG analysis showed the differential genes in different samples were significantly enriched in 22 pathways (*p*  <  0.05), including plant-pathogen interactions, plant hormone signal transduction, plant circadian rhythms, plant MAPK signaling pathway, alpha-linolenic acid metabolism, cysteine and methionine metabolism, and Photosynthesis (Tables S31 and S32, Figure S10). We also searched for homology of differentially expressed transcripts against the published *B. gymnorhiza* chloroplast genome [28], through BLAST (e-value < 1 × 10^−5^) and found two unigenes (TRINITY_DN16276_ c0_g24_i1 and TRINITY_DN11357_c0_g1_i1) which both are homologs of the gene paNDHK (NDHK_POPAL) that encodes the protein NAD(P)H-quinone oxidoreductase subunit K, that is involved in the BP category of oxidation-reduction process. Results of chloroplast blast are shown in the Tables S33 and S34.

### 2.6. Verification of RNA-Seq Data by qRT-PCR

**Figure 5:**
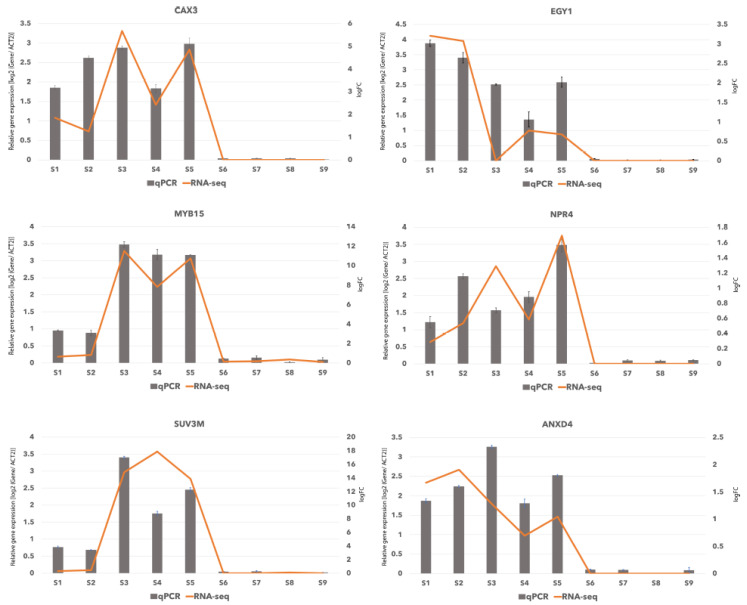
Verification of RNA-seq results by real-time quantitative PCR. Log_2_ value of the gene expression in oceanside/riverside for six selected genes. RNA-Seq results -log fold change (logFC)- are shown as bars and qRT-PCR results are shown as lines for relative gene expression [log_2_ (Gene/ACT2)].

The differentially expressed genes were functionally annotated (Table S24), and unigenes that appeared to be enriched in two BP categories of “response to salt stress: GO:0009651” and response to osmotic: GO:0006970 stress”, were then searched through BLAST against nucleotide and protein databases, to be assigned to their homologous genes in known species. Among the genes enriched in salt response category, there were 10 genes (Table S35), which 6 of them were shared with osmotic stress category and were upregulated in the oceanside according to RNA-seq analysis (Tables S21 and S35). We chose these 6 genes that encode proteins involved in salt and osmotic stress (Table S35), as representative genes to validate the data generated through RNA-Seq. They were subjected to quantitative real-time PCR (qRT PCR) and the primer pairs were designed using the unigene sequences obtained in this study. Real-time quantitative PCR (qRT-PCR) was performed for those six genes using aliquots of the same RNA samples that were used for RNA sequencing. The selected genes included BgCAX3 (homolog of AtCAX3: CATION EXCHANGER 3), BgEGY1 (homolog of AtEGY1: Ethylene-dependent, Gravitropism-deficient, and Yellow-green-like protein1), BgMYB15 (homolog of AtMYB15: MYB transcription factor), BgNPR4 (homolog of OsNPR4: NONEXPRESSER OF PR GENES 1 (NPR1)-like protein 4), BgSUV3 (homolog of Os SUV3 (suppressor of Var 3), BgANXD4 (homolog of AtANXD4: Annexin D4) (Table S35). The primer pairs were designed using unitranscript sequences obtained in this study by Primer3 (available online: http://primer3.sourceforge.net/) [29,30]. BgACT2 gene from the previous study of *B. gymnorhiza* [31], was used as a reference gene for internal control to normalize the qPCR efficiency as described previously [32,33]. Expression profiles for these genes deduced by qPCR revealed similar patterns to those from the RNA-seq analysis results and we found that all six genes were upregulated in oceanside samples (Figure 5). Although there were slight differences between RNA-seq data and qRT-PCR, the trend of selected genes measured by qRT-PCR was consistent with the results of transcriptome sequencing. Primers used for qPCR, and length of qPCR profucts are shown in Table S36.

## 3. Discussion

Although mangroves are one of the most ecologically important plants, until very recently no genomic resources were available for them, and their current genomic data is still very scarce. Therefore, the molecular mechanisms underlying high adaptability of mangroves in harsh, intertidal zone, and their tolerance to abiotic stresses is not well understood, mainly due to the little information in public databases about mangroves genomics and transcriptomic profiles. So far, there has only been one transcriptome study for the Rhizophoraceae mangrove family [23]. As it is known that species specific genomic data are dynamic and consist of both core genes which are the genes presenting in all individuals of one species, and dispensable genes that are genes that only exist in some individuals [11,34], therefore, a single genome or transcriptome assembly data might not represent the entire genomic information of one species. For instance, transposable elements cause variability in intergenic and/or genic regions, in both closely related species and individuals of the same species [34]. In this study, we have assembled and annotated a de novo transcriptome from the leaves of a mangrove tree species; *B. gymnorhiza*. We then analysed differentially expressed transcripts between two populations of this species that grow either in brackish water (riverside) or in saline water (oceanside) in their natural habitats where they show significant morphological differences (Figure 1, Table S1).

The number of retrieved unitranscripts in our study (132,268), was higher than that obtained in the previously reported transcriptome reconstructions in *B. gymnorhiza* [23]. The N50 length of the unitranscripts was 1786 bp, and the average length was 1150 bp with maximum length of 15,000 bp (Table S4). These numbers are also all higher than those of four mangrove genera in the same family (Rhizophoraceae), i.e., (*B. gymnorhiza*; N50 = 1374 bp, average length = 859 bp), (*kandelia obovata*; N50 = 1364 bp, average length = 838 bp), (*Rhizophora apiculata*; N50 = 1370 bp, average length = 858 bp), and (*Ceriops tagal*; N50 = 1317 bp, average length = 815 bp), and of one terrestrial species from this family; (*Carallia brachiata*; N50 = 1077 bp, average length = 715 bp). Additionally, the GC content of the assembled unitranscripts (43–45%) was slightly higher than that in previous mangrove study which ranged from 43.08% to 43.85% in the five Rhizophoraceae species [23]. When evaluating the consistency and completeness of the assembly, by mapping the reads back to the assembled transcriptome, the overall alignment rate was >95.46%, covering 98.61% of the reference sequences (Table S5) which is slightly more than that, reported for other mangrove species (93.04–96.48%) and much higher than that in other species such as *Arundo donax* [11], *Triticum *aestivum** [35], and in *Triticum *turgidum** [36] which was reported to be around ~70%. The quality of the *B. gymnorhiza* transcriptome obtained in this study was also comparable to, or better than those of most transcriptome assemblies listed in [37] and higher than that of the previous *B. gymnorhiza* transcriptome assembly (Table S6). Additionally, about 71% and 57% of the unitranscripts and predicted proteins were successfully assigned to genes by BLASTx and BLASTp searches against the UniProtKB/Swiss-Prot database (Table S9) which is similar to previous mangrove study and higher than that of another tree species; Downy Oak (*Quercus pubescens* Willd.) [38].

Organism distribution analysis based on BLASTx and BLASTp searches found *Arabidopsis thaliana*, *Oryza sativa*, and *Nicotiana tabacum* as the species that contained the most homologous genes with *B. gymnorhiza* (Figure S2, Tables S7 and S8). This was consistent with the previous study that also reported *A. thaliana* as the most homologous with *B*. *gymnorrhiza* [23]. In addition, the other most homologues species found in this study, such as *Ricinus communis*, *Populus trichocarpa*, *Vitis vinifera*, *Glycine max*, *Nicotiana tabacum*, and *Lotus japonicus* (Figure S2), were all reported to contain high number of homologous genes with the mangrove genome in a previous study [23]. In our study we found homology between *B. gymnorhiza* and *Lotus japonica* as well. In the previous study it was reported that *Carallia* *brachiate* had homologous hits with the genes in *Lotus japonicus*. *C.* *brachiata* is a non-mangrove species in the mangrove family (Rhizophoraceae) that has a basal position in phylogenetic tree according to molecular analysis [39]. Interestingly, this species also has developed aerial stilt roots, same as its mangrove relatives [40]. Finding homologous genes between *B. gymnorhiza* and *C.* *brachiata* could be related to the fact that *B. gymnorhiza* has a basal position in the phylogenetic tree of mangrove species in Rhizophoraceae family and therefore, is located the closest to the non-mangrove species Ca. *brachiata* [39].

When plants experience abiotic stress, they try to adapt to the stressful condition by adjusting the expression of a series of genes involved in complex networks. Salt stress is known to cause ionic, osmotic, and oxidative stress in plants. The high sodium ion levels generate cytoplasmic calcium signals [41]. Excessive salt ions decrease the osmotic potential of soil, and subsequently, the absorbing and discarding water will become challenging for the plants, which therefore leads to osmotic stress [42]. Response to osmotic stress is an important skill that plants learned in face of high salinity stress. Plants adjust ion concentrations inside and outside their cells via ion pumps imbedded in their membrane systems and accumulate small molecules such as sugars for osmotic pressure regulation [43]. Thus, plants have evolved to utilize several mechanisms in order to cope/adapt with the high salinity environment. These mechanisms include ion transport, ROS scavenging, osmotic regulation, and cell signal conduction [44,45]. In our study, the *B. gymnorhiza* GO analyses of transcriptome sequencing and DET data were found to be related to the genes involved in the above pathways.

According to the GO analyses, among transcripts that were upregulated in saline condition (oceanside plants), were those involved in the biological process of “response to salt stress” and “response to osmotic stress” (18 and 21 transcripts, from the gene category of GO:0009651 and GO:0006970 respectively) (Table S25), that were found to be homologous with genes such as *AtCAX3* which encodes a vacuolar cation/proton exchanger which translocate Ca^2+^ and other metal ions into vacuoles, and is involved in ion homeostasis [46], *AtEGY1* that encodes zinc metalloprotease EGY1 which is involved in the regulation of gene expression in response to ammonium stress and interacts with ABA signalling [47], *AtMYB15* that encodes transcription factor MYB15 which is involved in drought and salt tolerance [48], *OsNPR4* that encodes Ankyrin repeat-containing protein NPR4 which is involved in salt stress tolerance [49], *OsSUV3* that encodes ATP-dependent RNA helicase SUV3 which confers salinity and drought stress tolerances [50], *AtCIPK9* that encodes CBL-interacting serine/threonine-protein kinase 9, which is involved in K^+^ homeostasis under low-K^+^ stress [51], *AtANXD4* that encodes Annexin D4 which is involved in osmotic stress and abscisic acid signaling [52], *AtGASAE* that encodes a gibberellin-regulated protein which is involved in regulation of reactive oxygen species metabolic process [53], *AtCSCL1* that encodes a CSC1-like protein At1g32090 which acts as an osmosensitive calcium-permeable cation channel [54], *AtTTL3* that encodes an inactive TPR repeat-containing thioredoxin which is involved in osmotic and salt stress tolerance [55] (Table S35).

To overcome the osmotic stress from higher concentrations of Na^+^ in the vacuoles, plants accumulate compatible solutes in the cytoplasm, such as betaine, proline, free sugar, and polyalcohol. In our study, genes involved in ion transport and glycometabolism were identified. Among them, were glycosylhydrolase genes that participate in the glucose synthesis pathway in response to salt stress [13]. For example, TRINITY_DN75517_c1_g1_i1 was an upregulated transcript in the oceanside samples that was found to be homologous with *AtXTH7* that encodes a xyloglucan endotransglucosylase/hydrolase protein. Another example was the TRINITY_DN54214_c0_g3_i1, a homologous transcript for *AtPRP4* gene that encodes for proline-rich protein (Table S35). In addition, ion translocators on plants’ membrane systems, manage the excretion and intracellular segregation of extra salt ions and control ion homeostasis under salt stress. Transporters, located in the plasma membrane, cytoplasm, or nucleus, are involved in several signalling pathways [56]. In our study, many transcripts that were upregulated in saline condition (oceanside samples) were involved in the biological process of ion (including manganese, zinc, phosphate, sulphate, potassium) homeostasis and ion transport that are homologous with genes such as *AtCCX1* encoding cation/calcium exchanger that acts as a vacuolar membrane-localized H^+^-dependent K^+^ and Na^+^ transporter [57], and *AtCNGC2* encoding cyclic nucleotide-gated ion channel 2 [58] (Table S35).

Stomatal development, and movement mechanisms are well regulated in plants, in order to control transpiration and water use efficiency (WUE). TRINITY_DN86770_c0_g1_i6 was an upregulated transcript from the GO biological process of regulation of stomatal complex development” (GO:2000122). This transcript was found to be homologous with the gene *AtBCA1* which encodes Beta carbonic anhydrase that promotes water use efficiency and is involved in the CO_2_ signalling pathway that controls gas exchange between plants and the atmosphere by modulating stomatal development and movements [59,60]. Another transcript that was upregulated in saline condition was TRINITY_DN68991_c0_g1_i2 (the GO biological process of “negative regulation of DNA methylation”: GO:1905642) which is a homolog of *AtASI1* genes (Tables S25 and S34). This gene which is also known as *IBM2* [61] encodes a protein named ANTI-SILENCING1 that is required to prevent promoter DNA hypermethylation and transcriptional silencing of some transgenes [62]. It is reported to play a similar role to that of the histone H3K9 demethylase *JMJ25/IBM1* in preventing CHG methylation in the bodies of numerous genes and ensuring the proper expression of *JMJ25/IBM1* full-length transcript [61]. The upregulation of this transcript in the saline condition might imply the role of epigenetic modifications in regulation of stress-response genes in mangrove trees. On the other hand, the GO molecular function of “methyltransferase activity”: GO:0008168 was enriched in downregulated transcripts in the oceanside samples (Table S29). Gene ontology terms related to methylation (GO:003225), chromatin modification (GO:0006325), RNA modification (GO:0009451) and methyltransferase activity (GO:0008168) were retrieved in downregulated transcripts in saline condition as well (Table S28). Future studies are needed to investigate the role of DNA methylation and other possible epigenetic marks in adaptation of mangrove species.

High salinity can not only cause osmotic stress in plants, but can also accumulate excessive reactive oxygen species (ROS), and induce oxidative stress, which can further prevent normal metabolism in cells, and therefore result in oxidative damage and even death [63,64]. Plants use proper regulation of oxidative stress response genes in order to ensure the removal of intracellular ROS, thus reducing oxidative damage of high salinity [43]. In our study, many transcripts that were upregulated in saline condition (oceanside samples) were involved in the biological process of oxidation-reduction process (GO:0055114). These transcripts were found to be homologous with genes such as *PER4_VITVI* encoding a peroxidase that is involved in response to oxidative stress [65], *C93A1_SOYBN* encoding 3,9-dihydroxypterocarpan 6A-monooxygenase which is a cytochrome P450 involved in the biosynthesis of the phytoalexin glyceollin [66], *AtPER25* encoding peroxidase_25 protein that is involved in removal of H_2_O_2_, oxidation of toxic reductants, biosynthesis and degradation of lignin, and response to environmental stresses such as oxidative stress [67] (Table S35). In addition, when transcripts in our study were searched against the published *B. gymnorhiza* chloroplast genes through BLAST, two homologs were found to be the chloroplast gene named *paNDHK* that encodes NAD(P)H-quinone oxidoreductase subunit K and involves in the BP category of oxidation-reduction process (Table S35).

Furthermore, in our transcriptome study, many homologous transcripts encoding for lignin, and cellulose were also retrieved (e.g., cellulose catabolic process (GO:0030245), and cellulose metabolic process (GO:0030245)) (Table S25). Many transcripts involved in cell wall organization (GO:0071555) were found upregulated in saline condition as well. These transcripts showed to be homologous with genes such as *AtCSLC5* encoding xyloglucan glycosyltransferase that is a β-1,4-glucan synthase rather involved in the synthesis of the xyloglucan backbone than cellulose. Xyloglucan is a non-cellulosic polysaccharide of plant cell walls and consists of a glucan backbone substituted by xylose, galactose and fucose [68]. Cellulose and lignin make secondary wall in plants which is an important defence mechanism against abiotic stresses.

Apart from these, transcription factors which are regulatory proteins that bind to specific nucleotide sequences upstream of genes and either induce or repress the expressions of those targeted genes, are also essential components in the signalling network, and have been implicated in the regulation of plant stress response [43,69,70]. Members of the transcription factor family such as *bZIP*, *WRKY*, *AP2/EREBP*, *C_2_H_2_*, *bHLH*, *MYB*, *NAC*, and *C_2_H_2_-Dofandthese* are reported to be involved in the plant response to salt stress [71]. In our study many of these transcription factor genes which are related to response to salt stress were identified, such as transcription factors from *AP2, bZIP, WRKY, NAC*, and *MYB* families (Tables S11 and S12), suggesting that these transcription factors may play a regulatory role in response to salt stress in *B. gymnorhiza* and possibly in other mangrove species. 

Biological pathways analyses play an important role in understanding functional role of transcriptomic data. KEGG (available online: http://www.genome.jp/kegg/) is a highly integrated database of biological systems that combine genomic, chemical and systemic functional information [72]. In our study one of the pathways that was enriched in oceanside plants, was mitogen-activated protein kinase (MAPK) pathway (Table S31). Plants have large families of MAPK pathway components that their activation has been reported in response to abiotic stimuli such as salt, drought, cold, heat, and wounding in plants [73]. In addition, purine metabolism has been reported to be involved in activating plant abiotic defences. A study using *Arabidopsis oxt1* mutants has shown that changes in cellular adenine levels lead to increased stress tolerance and increased biomass in plants, suggesting a link between purine metabolism, plant growth, and stress acclimation [74]. In our study purine metabolism pathway was upregulated in oceanside samples (Table S31). Furthermore, allantoin is a metabolic intermediate of purine catabolism that often accumulates when plants subjected to abiotic stress. It activates jasmonic acid responses via ABA which is shown in *Arabidopsis* knockout mutants (*aln*) of *ALLANTOINASE*, suggesting the potential role of allantoin in interaction between purine catabolism and stress phytohormone signalling [75]. In our transcriptome analyses, homologous transcripts encoding for *ALN* genes were retrieved (Table S19). The presence of homologous transcripts to genes involved in the abovementioned pathways and in the biological process, provides new insights into molecular mechanisms underlying the extreme adaptability and robustness of *B. gymnorhiza* to high stress environmental condition, thereby may contribute to mangrove’s importance as ecologically virtual trees. The identified homologous transcripts involved in key metabolic pathways provide a platform for directing future efforts in genetic improvement of mangroves and other tree species under stress.

Our results are consistent with previous salt stress studies in other plants. For example, a transcriptome analysis of a salt-tolerant cotton cultivar (*Gossypium hirsutum*) under temporal salt stress, has identified differentially expressed genes that -similar to our study- were mainly related to stress pathways such as “response to oxidative stress”, “response to salt stress”, “response to water deprivation”, “ion transport”, and “metal ion transport”. Differently expressed genes related to ion homeostasis, transcription factors and cell wall modification were also found highly active in response to salt stress in cotton same as our study [76]. Studying transcriptome changes under salt stress in *Populus wutunensis* which is a salt and drought tolerant species, differentially expressed genes were identified in salt treated leaves that were also involved in ion transport, osmotic regulation, and reactive oxygen species (ROS) scavenging [13]. In another study, gene expression analyses in *citrus limonia* which is a salt tolerant crop species, revealed upregulation of salinity-induced genes, that were mainly associated with stress response, ion transport, and signalling, regulation of stomatal movement, and oxidative stress [15]. Another transcriptome and gene expression analysis in salt tolerant *Suaeda salsa* after salt stress treatment, identified DEGs involved in ion transport, reactive oxygen species (ROS) scavenging. Same as our study transcriptional factors were also expressed upon salt treatment [77].

In summary, our study provides a good quality publicly available leaf transcriptome for *B. gymnorhiza*, a representative species of highly adapted mangrove trees. The transcriptomic data generated in this study provide invaluable resources to understand the biology of mangrove trees, and may help with developing stress resistant, productive, and economically sound genotypes in other plant species. The functional annotation of the transcriptome would add benefits to providing insights into the molecular mechanisms underlying mangroves’ extreme adaptability. It should be of value to the future functional genomics and genetic studies for not only mangroves but other tree species growing under stressed condition. Although our study is based only on one vegetative tissue (leaf), it has broadened our knowledge of the transcriptome profile in *B. gymnorhiza* trees in their natural environment. It should also be noted that, in our study we focused on one of the main stress factors in mangrove habitats (salinity), as it has been reported to have major effect in plants’ life [5], and it is remarkably at high level in mangroves habitats. Future studies are needed to focus on other stress factors along with salinity and evaluate how they may affect the adaptation of mangrove trees and possibly other tropical plants.

## 4. Materials and Methods

### 4.1. Plant Material, Soil Salinity and Morphological Measurements

One new born fresh leaf from the tip of a branch from each of the nine mature individuals of *B. gymnorhiza* (*Rhizophoraceae*) trees (five biological replicates from the oceanside and four from the riverside), in a mangrove forest located along the estuary of the Okukubi River, Okinawa Island—Japan, and the nearby coastal area of Pacific Ocean—Japan (26°27′ N, 127°56′ E), were collected, frozen in liquid nitrogen, and stored at −80 °C for RNA extraction. The riverside trees were located along the estuary of the river, and the oceanside trees grew along the coastal area of the Japanese Pacific Ocean. The sampling was performed from 12:30–13:30 on the 28 June 2019 from both oceanside and riverside plants. The salinity of soil water surrounding the roots was measured using a Refractometer W/ATC 300011, SPER Scientific (Scottsdale, AZ, USA) and was recorded as 3.5% at the oceanside and 2.0% at the riverside. Morphological traits were measured on 30 randomly-chosen *B. gymnorhiza* individuals (15 from the oceanside and 15 from the riverside). We measured tree height, and tree diameter at breast height (DBH). We also collected 15 leaves from the distal end of lower branches of those 30 measured trees in order to measure leaf length, and leaf width. The results were tested for significant differences between sites using the Welch Two Sample *t*-test in R software (available online: http://www.R-project.org—version 3.5.0—April 2018) (Table S1).

### 4.2. RNA-Seq and Transcription Analyses

#### 4.2.1. RNA Extraction

A total of nine Hi-Seq libraries of RNA samples (five of oceanside and four of riverside plants) were sequenced and analysed. Total RNA isolation was performed by ada pting the method described in [78], which combined a CTAB-based lysis solution with silica column-based RNA binding, DNase treatment, and washing steps using RNeasy Plant Mini Kit (Qiagen-, Hilden, Germany). RNA concentration was determined using Qubit (Qubit QC, Thermo Fisher Scientific, Waltham, MA, USA). Total RNA was treated with RNase-free DNase I (Takara Bio Inc. Japan) to remove DNA. The integrity of RNA samples was evaluated using an Agilent 2100 Bioanalyzer (Agilent Technologies- (Agilent Technologies Canada, Inc., Mississauga, ON, Canada)), and only high-quality RNA samples (RNA integrity number ≥ 8.0) were used for RNA-Seq library construction.

#### 4.2.2. Library Construction and Sequencing

RNA samples were submitted to OIST sequencing centre for a second RNA quality checking, library preparation, and paired-end mRNA-sequencing (PE mRNA-seq). Briefly. Library preparation and sequencing the cDNA library for RNA-Seq was constructed using TruSeq™ RNA Library Prep Kit (Illumina, San Diego, CA, USA) (Catalogue No. RS-122-2201) according to the manufacturer’s instructions, and was then sequenced using Next Seq (Illumina Inc.) to obtain ~150 bp sequences from both ends of each cDNA with an insert size of 300–400 bp. After sequencing, quality control of raw RNA-Seq reads was performed using FastQC v0.11.3 (available online: http://www.bioinformatics.babraham.ac.uk/projects/fastqc/). Raw Illumina RNA-seq data from all libraries were trimmed for quality using Trimmomatic-0.36 [79]. Illumina sequence adapters, leading low quality (below quality 3) were removed; N base pairs were trimmed, and the resulting trimmed reads were scanned using a 4-bp sliding window and were cut when the average quality per base dropped below 30. Read pairs where both reads were ultimately of at least 36 base pairs in length following this quality control process were retained and used for subsequent analyses. All the Illumina sequencing reads generated in this study are deposited in the NCBI PRJNA754249.

#### 4.2.3. De Novo Transcriptome Assembly

Filtered reads were used for transcriptome assembly using Trinity pipeline (version 2.4), with the default parameters (*K* = 25 and a minimum transcript length of 200 bp) [19]. The longest isoform for each gene was selected (available online: https://groups.google.com/forum/#!topic/trinityrnaseq-users/cXM1KiJe7dU). Clustering of redundant transcripts was performed with 95% identity and a word size of 10, using CD-HIT v4.6.4 [20]. The de novo assembled transcripts were then used as a reference for mapping the individual reads back to it, and estimating transcript abundance using RSEM (RNA-Seq by Expectation-Maximization) [26]. We then filtered out any transcripts with less than 1% of the per-component expression level (IsoPct) using a script bundled with Trinity. These transcripts with low support are likely to be transcript assembly artifacts. The quality and completeness of the de novo assembly were further evaluated using BUSCO-3.0.2 analysis [22]. This quality assessment tool provides high-resolution quantifications for genomes, gene sets, and transcriptomes and checks whether each of the BUSCO group is complete, duplicated, fragmented, or missing in the genome or transcriptome assembly. The leaf unitranscripts were compared to the set of Embryophyta genes, which contains 1440 BUSCO groups from a total of 31 species in order to obtain a quantitative measure of the transcriptome completeness, based on evolutionarily informed expectations of gene content from near-universal single-copy orthologs [22]. In addition, to evaluate the assembly consistency and alignment rate, the filtered unique reads were mapped back to the final assembled transcriptome using the alignment software Bowtie2 (available online: https://sourceforge.net/projects/bowtie-bio/). All analyses were operated using OIST-Linux-based supercomputer clusters (2 Intel Xeon E5-2600 v3—48 threads and 128 GB memory).

### 4.3. Functional Annotation of the Transcriptome

Assembled unitranscripts were used as input to predict potential coding sequences and identify open reading frames (ORFs) using TransDecoder-3.0.1 (available online: http://transdecoder.github.io) with default parameters [19]. After ORFs were extracted from the assembly, Trinotate pipeline was used to carry out comprehensive functional annotation of the transcripts leveraging various annotation databases (eggNOG/GO/KEGG databases) (available online: http://trinotate.github.io) [19]. Both nucleotide transcripts and protein sequences were used to search against the UniProtKB/Swiss-Prot (uniprot_sprot.trinotate_v2.0.pep.gz) and UniRef90 (uniprot_uniref90.trinotate_v2.0.pep.gz) databases using NCBI-BLASTx and BLASTp v2.2.28+ (e-value 1 × 10^−5^—max_target_seqs 1 -outfmt 6), respectively. The UniProtKB is a collection of functional information on proteins, with accurate, consistent, and rich annotation; the section Swiss-Prot contains manually annotated records. The UniRef databases provide NR clustered sets of sequences from the UniProtKB (including isoforms) and the UniProt Archive (UniParc) records (a comprehensive and NR database that contains most of the publicly available protein sequences). Predicted proteins were annotated using profile hidden Markov models with HMMER (v3.1b2) [80], against Pfam-A databases [81]. Based on these annotations, Gene Ontology (GO), Pfam and Kyoto Encyclopedia of Genes and Genomes (KEGG) terms were assigned to each unigene. In addition, prediction for signal peptides, transmembrane domains and rRNA transcripts was conducted by SignalP (v4.1) [82], TMHMM (v2.0) [83] and RNAMMER (v1.2) [84], respectively. Finally, all annotations were loaded into the Trinotate SQLite database, and a final annotation report was generated. The maximum e-value for reporting the best hit and associated annotation was 1 × 10^−5^.

### 4.4. Transcript Abundance and Differential Expression Analysis

To quantify transcript abundance, we applied the alignment-based methods contained in the Trinity package, by mapping the reads of each biological replicate against the assembled transcriptome. This was obtained with the align_and_estimate_abundance Perl script. In this analysis, RSEM (available online: http://deweylab.github.io/RSEM/package) [26] was used as the abundance estimation method and bowtie2 (available online: https://sourceforge.net/projects/bowtie-bio/) was chosen for the alignment by mapping the raw reads onto the assembled transcriptome. When the transcript abundance for each biological replicate had been obtained, we built a Gene Expression Matrix using the abundance_estimates_to_matrix.pl script to generate a normalized expression values matrix that was used to construct a matrix of counts and a matrix of normalized expression values. PtR script was used to generate correlation matrix and principal component analysis (PCA) plot for comparing replicates across all the samples. Differentially expressed genes (DEGs) among the oceanside and riverside samples libraries from the count matrix using run_DE_analysis.pl were found by using the Empirical Analysis of Digital Gene Expression (edgeR) (available online: http://biocon-ductor.org/packages/release/bioc/html/edgeR.html) statistical package [27]. The analyze_diff_expr.pl script was used to examine GO enrichment and to extract all transcripts that had *p*-values at most 1 × 10^−3^ and were at least 2^2^-fold differentially expressed. The differentially expressed (DE) features were partitioned into clusters with similar expression patterns by define_clusters_by_cutting_tree.pl script with Ptree method. The normalization factors were calculated using trimmed mean of M-values (TMM) method. The threshold FDR  <  0.05 was adjusted to identify the differentially expressed genes by fold change (≥2). We then used the David Gene Ontology (available online: https://david.ncifcrf.gov/home.jsp) for functional annotation of expressed homologous gene pairs to determine overrepresented GO categories across biological processes, cellular components, and molecular function domains. Enrichment of GO terms was tested using Fisher’s exact test, with *p*  < 0.05 considered as significant. The protein sequences were also searched against the KEGG database for KEGG Orthology (KO) assignments and pathway annotation. GO enrichment sets were further summarized using ReviGO (available online: http://revigo.irb.hr/) [25]. This program removes redundant GO terms and the similarity between terms is reflected by semantic space. For ReviGO analysis, the *Arabidopsis thaliana* and *Oryza sativa* databases were selected as GO term size using SimRel [85] as a standard for semantic similarity measurement. The output data of all GO annotations were thoroughly examined to identify those involved in stress.

### 4.5. Validation of Differentially Expressed Genes by qRT-PCR

The differential expressed genes were functionally annotated (Table S24), and unigenes that appeared to be enriched in two BP categories of “response to salt stress: GO:0009651” and response to osmotic: GO:0006970 stress”, were then searched through BLAST against nucleotide and protein databases and were assigned to their homologous genes in known species. Among the genes enriched in salt response category, there were 10 genes, which 6 of them were shared with osmotic stress category that were shown with RNA-seq analysis to be upregulated in the oceanside samples (Tables S21 and S35). We chose these 6 genes that encode proteins involved in salt and osmotic stress (Table S35) as representatives to validate the data generated through RNA-Seq. They were subjected to quantitative real-time PCR (qRT PCR) and the primer pairs were designed using the unigene sequences obtained in this study that were assigned to these stress response genes. RT-qPCR was carried out using aliquots of the same RNA samples that were used for RNA sequencing on a Thermocycler qPCR machine (BioRad, Hercules, CA, USA). Two micrograms (2 μg) of total RNA were used for complementary DNA (cDNA) synthesis by Prime Script II reverse transcriptase (TAKARA) using an oligo (dT) primer. ACT2 gene was used as a reference, internal control gene for normalization. The cDNA was diluted 5- to 10-fold, and the qPCR reactions were carried out in duplicate (with two technical replicates), using Takara SYBR Premix Ex Taq II (Takara Bio Inc.) and incubated at 95 °C for 3 min followed by 40 cycles of 95 °C for 15 s, 58 °C for 15 s and 72 °C for 15 s. Primer pairs were designed using Primer3 (available online: http://primer3.sourceforge.net/) [29,30]. Parameters for the primer design were as follows: minimum, maximum, and optimal sizes were 18, 24, and 20 nt; minimum and maximum GC contents were 40 and 60%; and minimum and maximum *T*_m_ values were 52 and 63 °C, respectively. The primer sequences for the unigenes are provided in Table S36. PCR specificity was checked by melting curve analysis, and the expression levels were calculated using the 2^−∆∆Ct^ method [86]. Data were analyzed and plotted using Microsoft Excel 2010.

## Data Availability

All the Illumina sequencing reads generated in this study are deposited in the NCBI PRJNA754249.

## Supplementary Materials

Supplementary materials are available online at https://www.mdpi.com/article/10.3390/ijms22189874/s1.
