# Supplementary material for: De Novo Transcriptome Assembly, Functional Annotation, and Transcriptome Dynamics Analyses Reveal Stress Tolerance Genes in Mangrove Tree (Bruguiera gymnorhiza)
Source: Int J Mol Sci. 2021 Sep 13;22(18):9874. doi: 10.3390/ijms22189874 (PMC8467813; doi:10.3390/ijms22189874)
Supplement: Supplementary file 1 [file ijms-22-09874-s001.zip › Supplementary_figures.pdf]

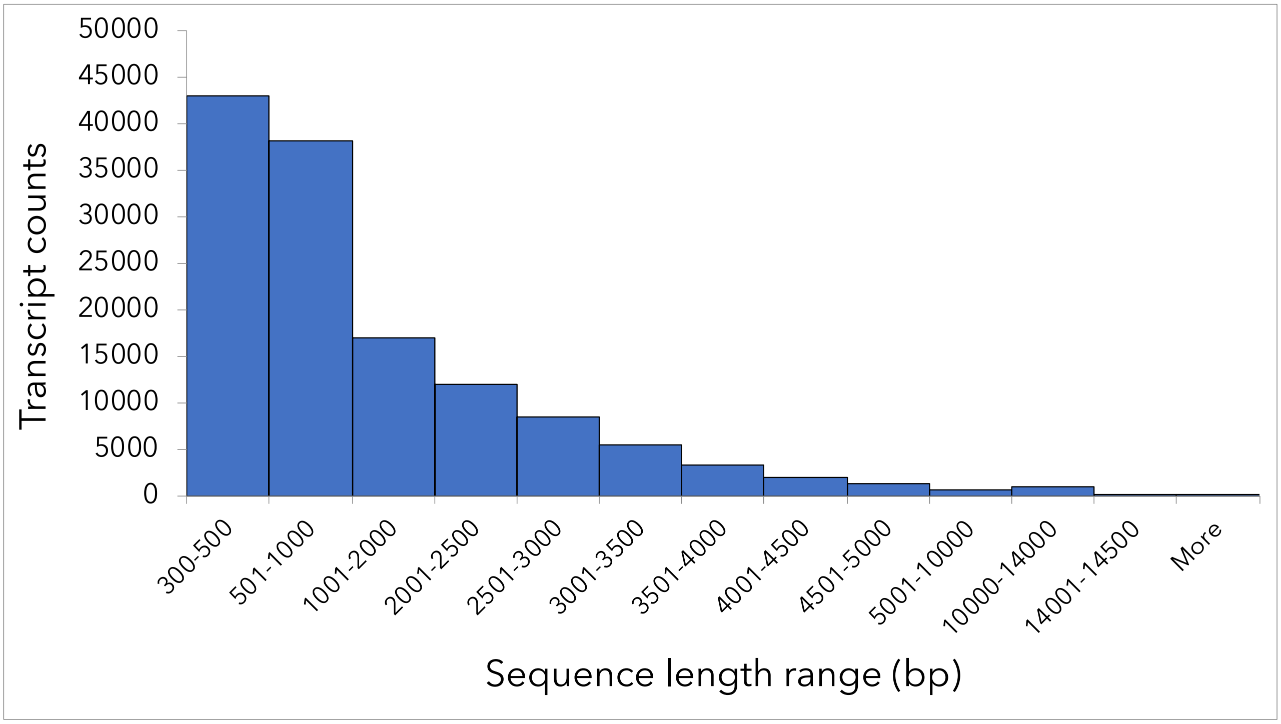

Figure S1. Sequence length distribution of non-redundant (NR) unique unitranscript sequences after sequencing and de novo assembly of *B. gymnorhiza* leaf transcriptome. The *X*-axis represents the length range bins in bp. The Y-axis represents the frequency of transcripts in each bin.

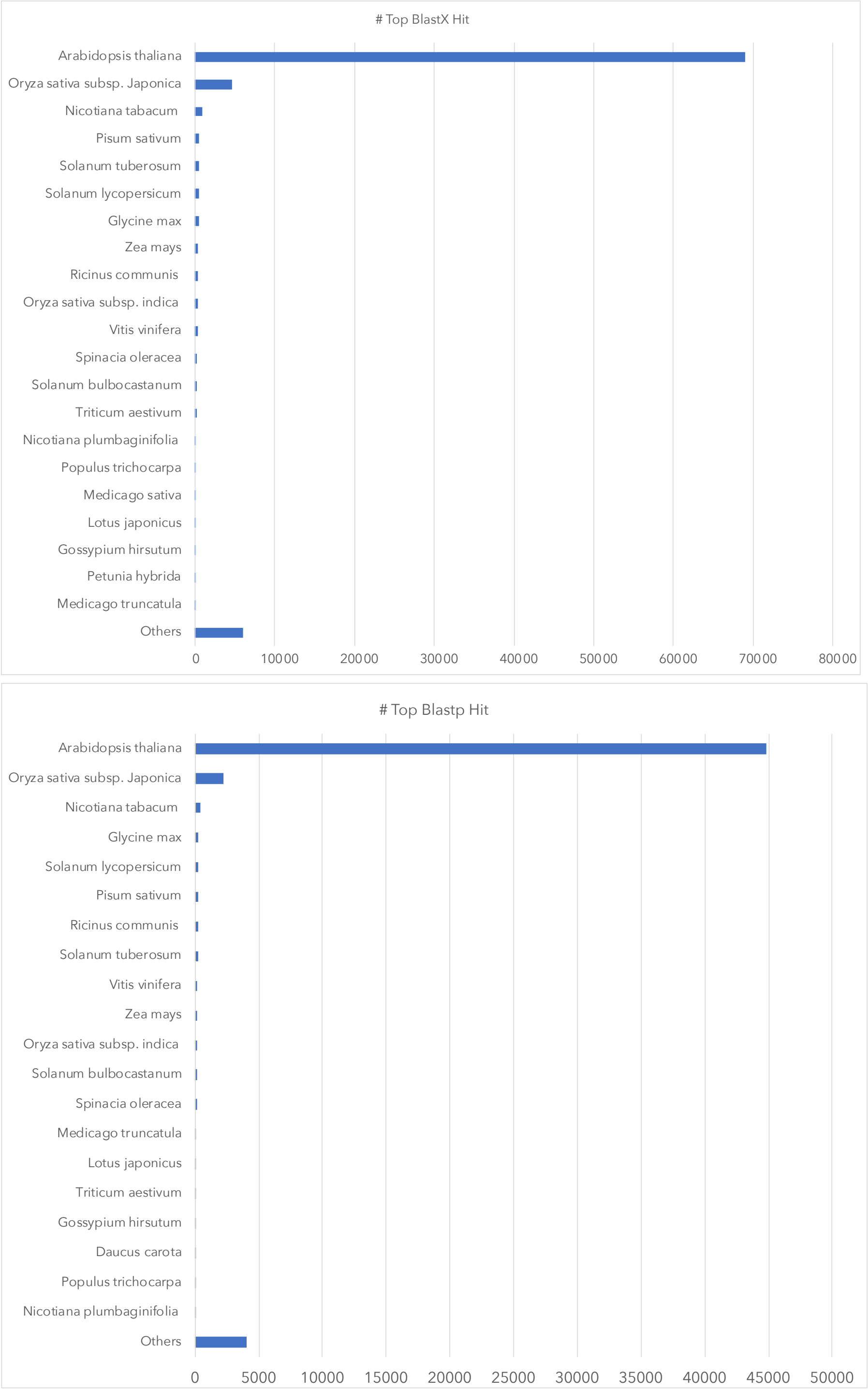

Figure S2. BLAST Top-Hits species distribution for *B. gymnorhioza* leaf transcriptome when compared with nr database (Top), and when compared with Swiss-Prot database (Bottom).

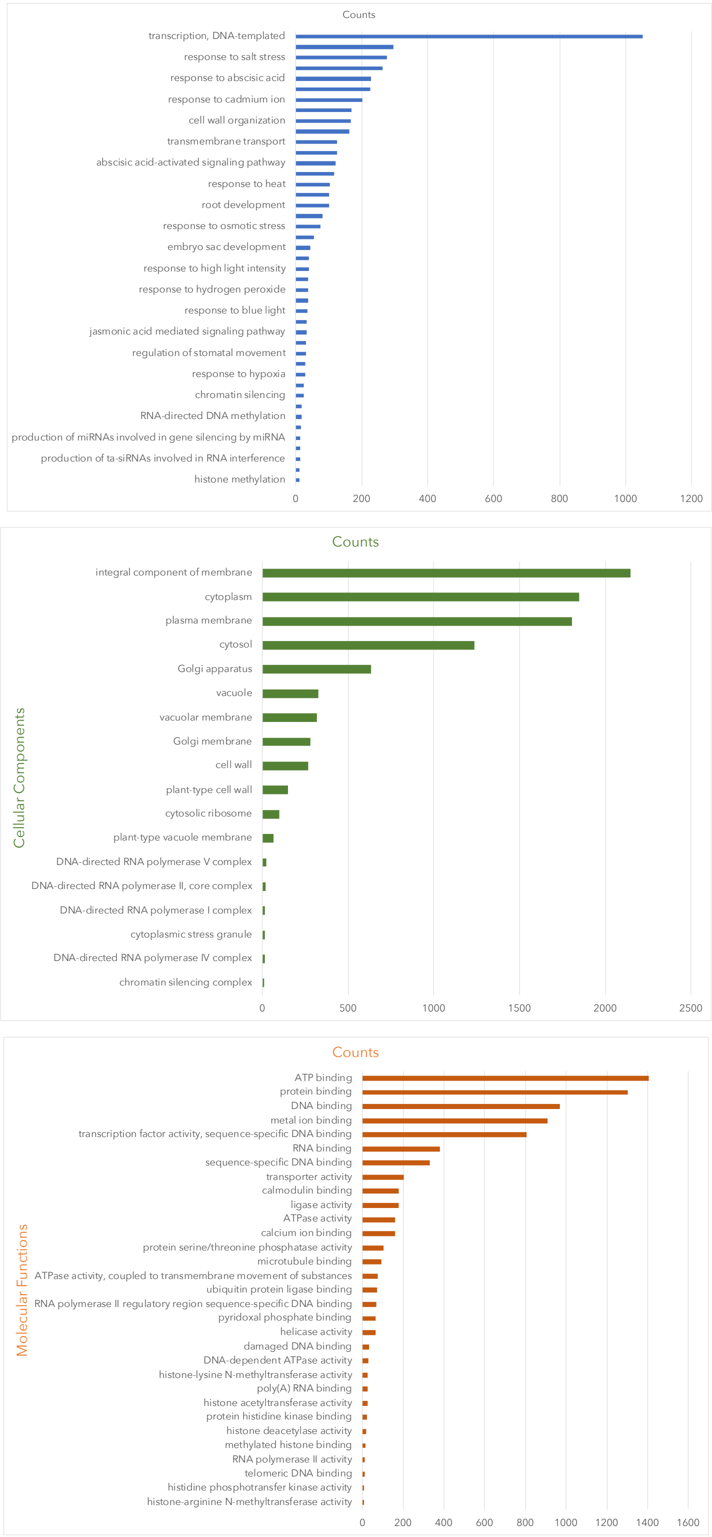

Figure S3. Gene ontology (GO) functional classification of *B. gymnorhiza* leaf transcriptome BLAST Top-Hits species distribution when compared with nr database. Histograms of the frequency of transcripts annotated to specific GO categories; biological process, cellular components, and molecular functions are represented by blue, green, and orange bars, respectively.

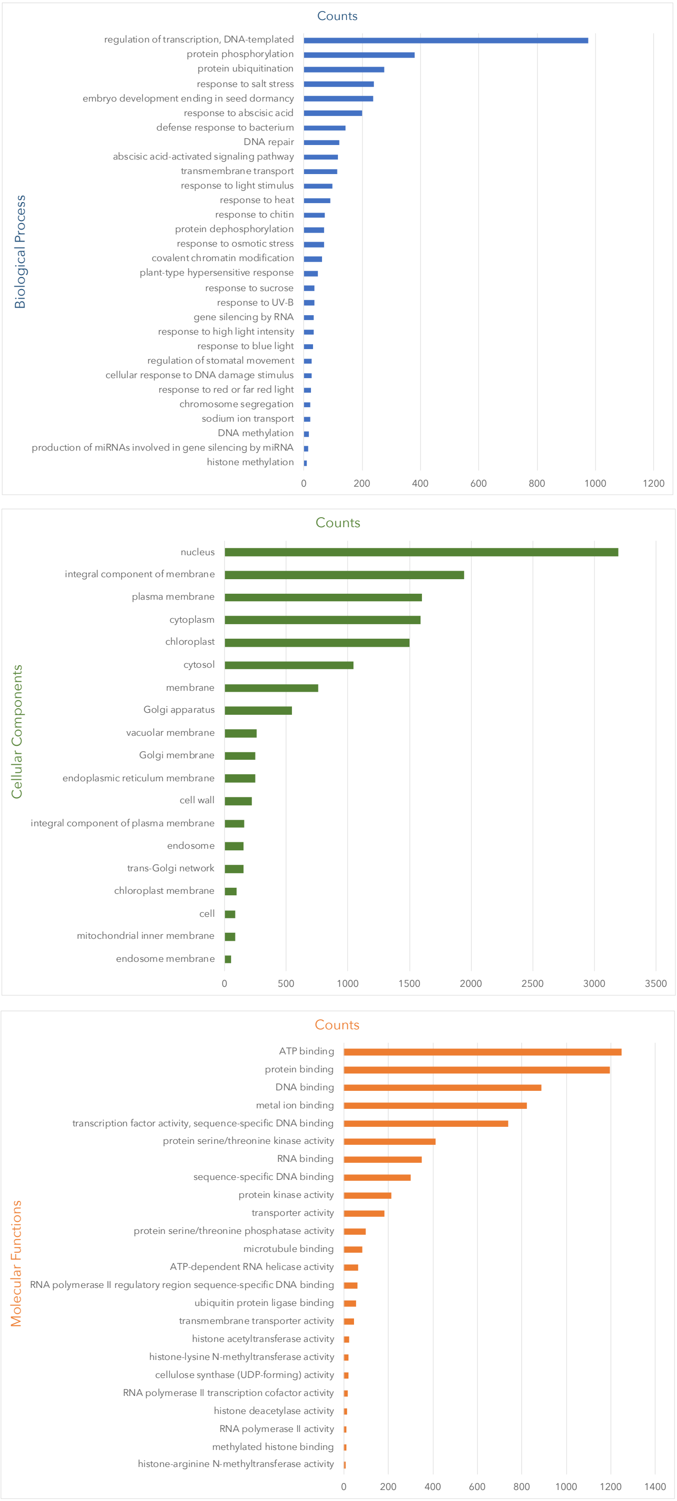

Figure S4. Gene ontology (GO) functional classification of *B. gymnorhiza* leaf transcriptome BLAST Top-Hits species distribution when compared with Swiss-Prot database. Histograms of the frequency of transcripts annotated to specific GO categories; biological process, cellular components, and molecular functions are represented by blue, green, and orange bars, respectively.

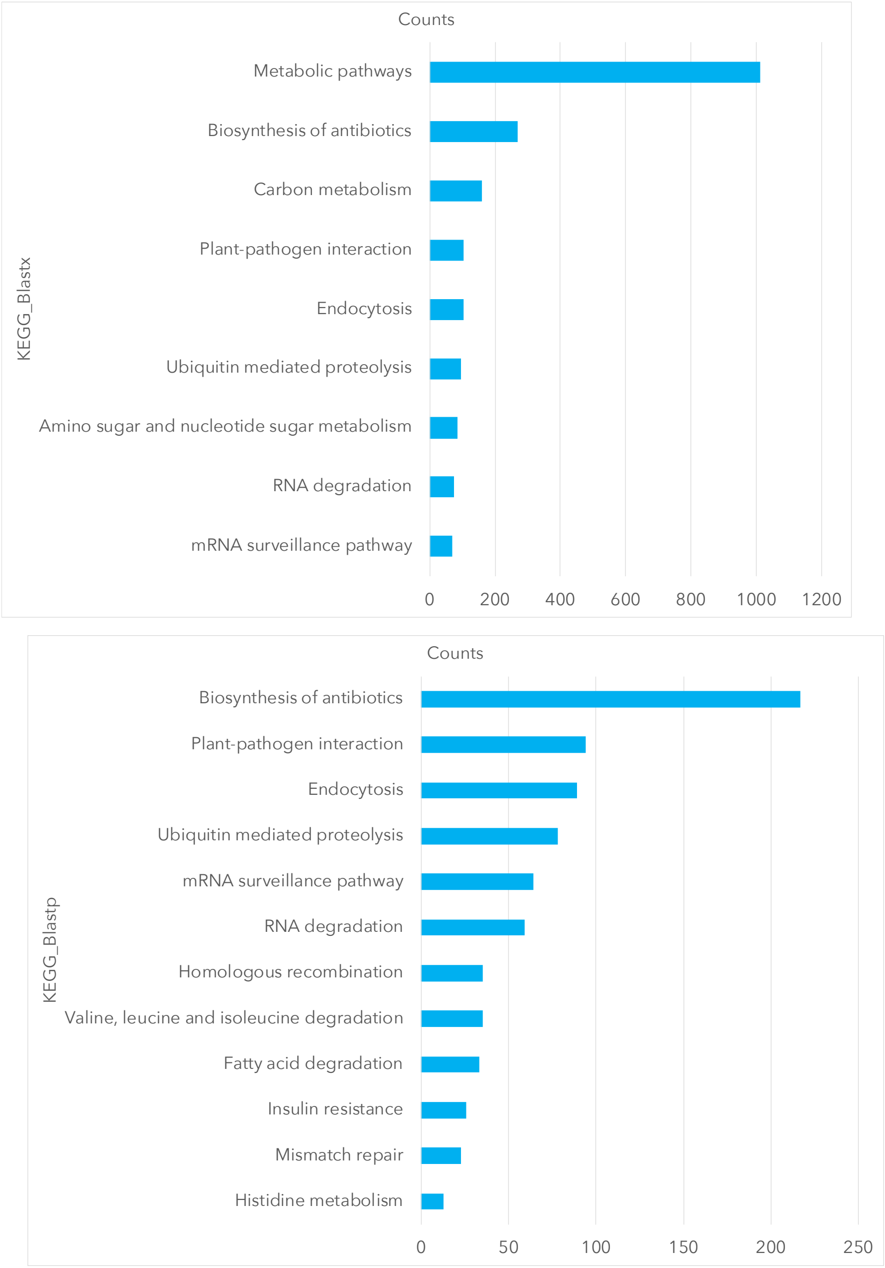

Figure S5. Kyoto Encyclopedia of Genes and Genomes (KEGG) analysis of *B. gymnorhiza* leaf transcriptome BLAST Top-Hits species distribution when compared with nr database (top) and Swiss-Prot database (bottom). Histograms of the frequency of transcripts annotated to KEGG pathways.

BP_Enriched

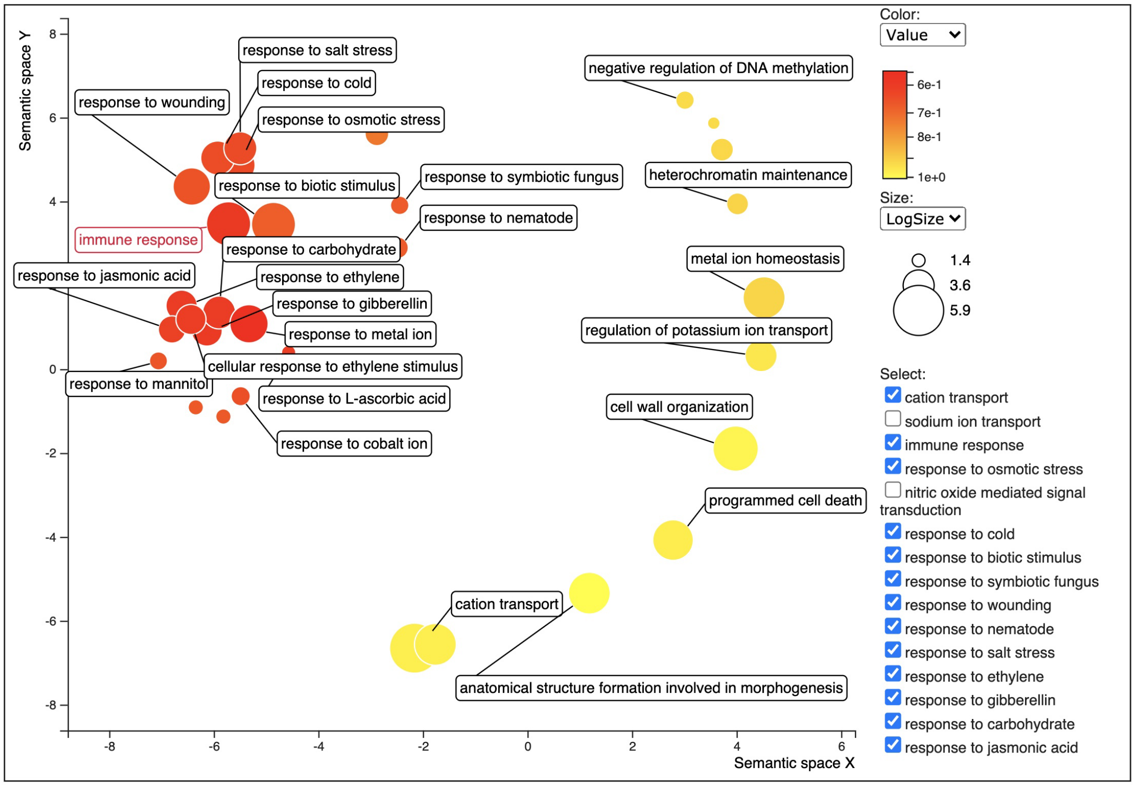

Figure S6. Biological process category of Gene Ontology (GO) enrichment analysis for upregulated genes in oceanside samples using REVIGO. Circles in closer proximity have more closely related GO terms. The size of the circles indicates the number of GO terms. The colour of the circle represents the significance of the enriched GO terms.

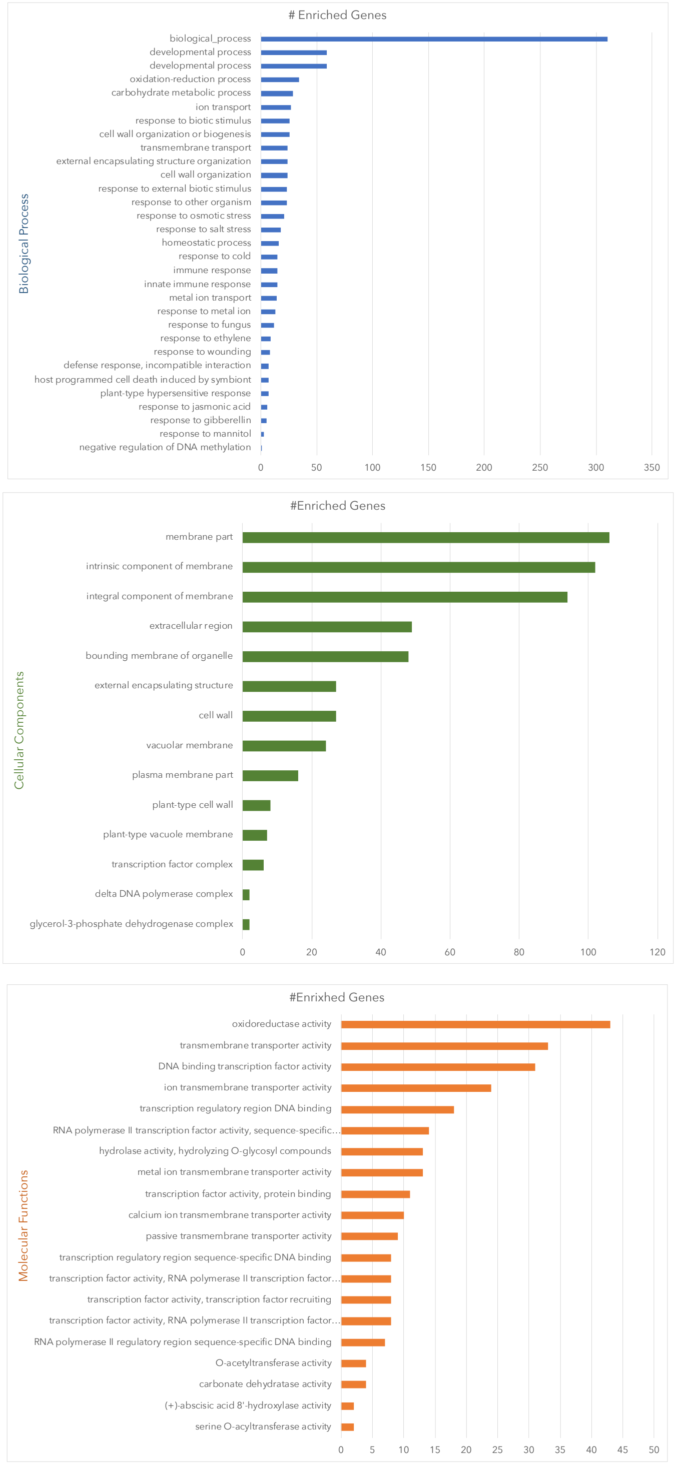

Figure S7. Biological process category of Gene ontology (GO) functional classification of upregulated transcripts BLAST Top-Hits species distribution when compared with Swiss-Prot database. Histograms of the frequency of transcripts annotated to specific GO categories; biological process, cellular components, and molecular functions are represented by blue, green and orange bars, respectively.

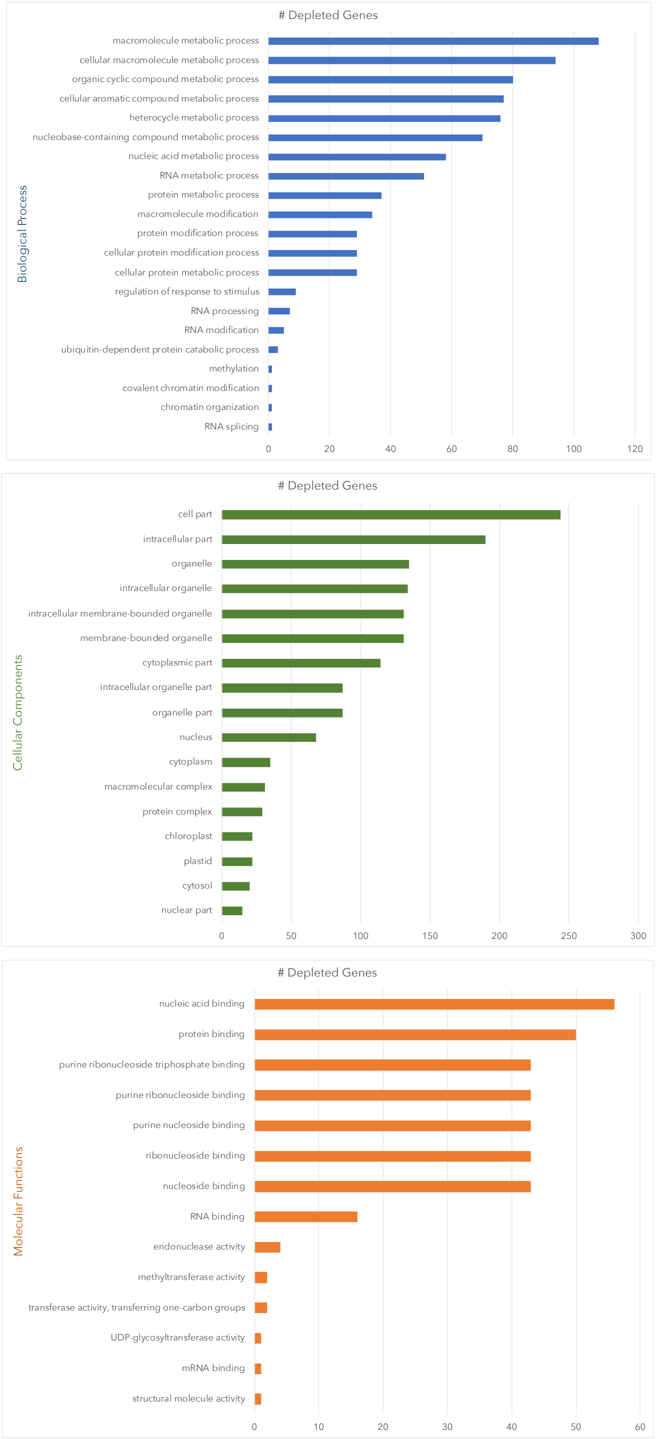

Figure S8. Biological process category of Gene ontology (GO) functional classification of upregulated transcripts BLAST Top-Hits species distribution when compared with Swiss-Prot database. Histograms of the frequency of transcripts annotated to specific GO categories; biological process, cellular components, and molecular functions are represented by blue, green, and orange bars, respectively.

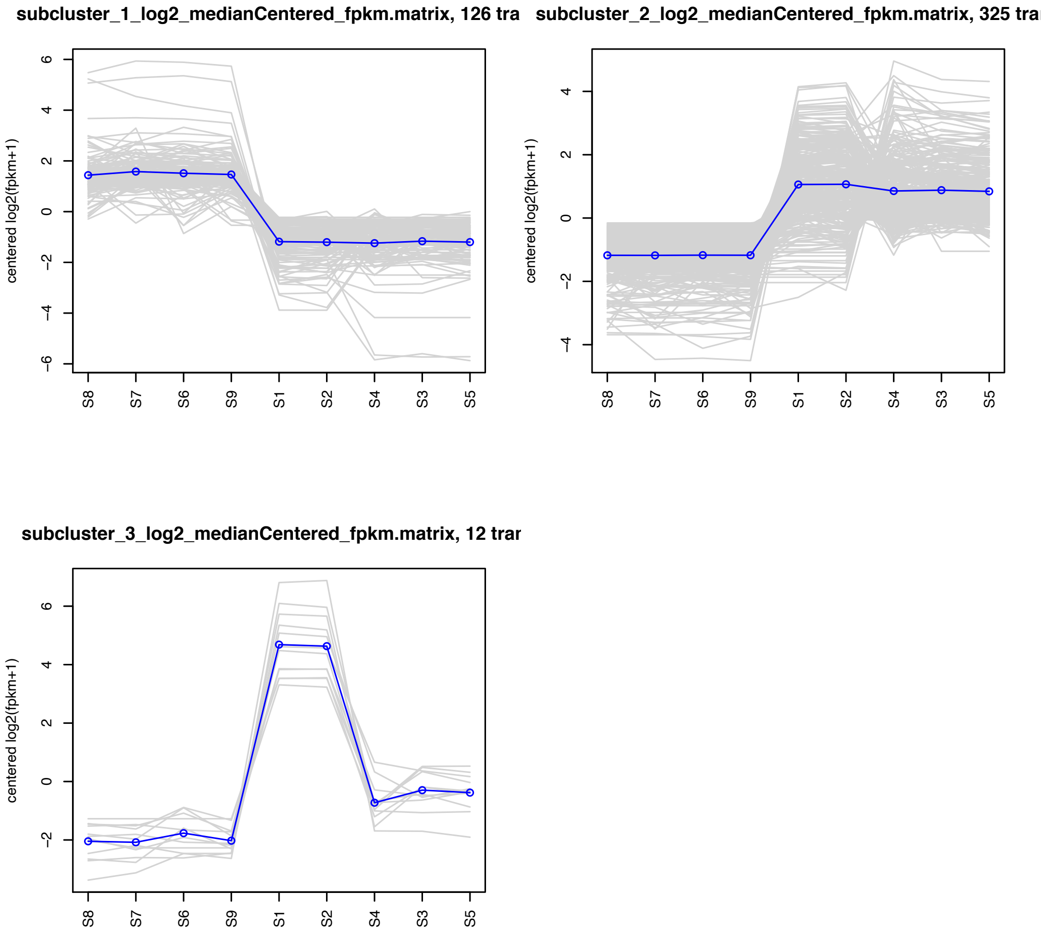

Figure S9. Transcript clusters extracted from the hierarchical clustering with R. *X*-axis: samples; y-axis: median-cantered log2 (FPKM). Grey lines, individual transcripts; blue line, average expression values per cluster.

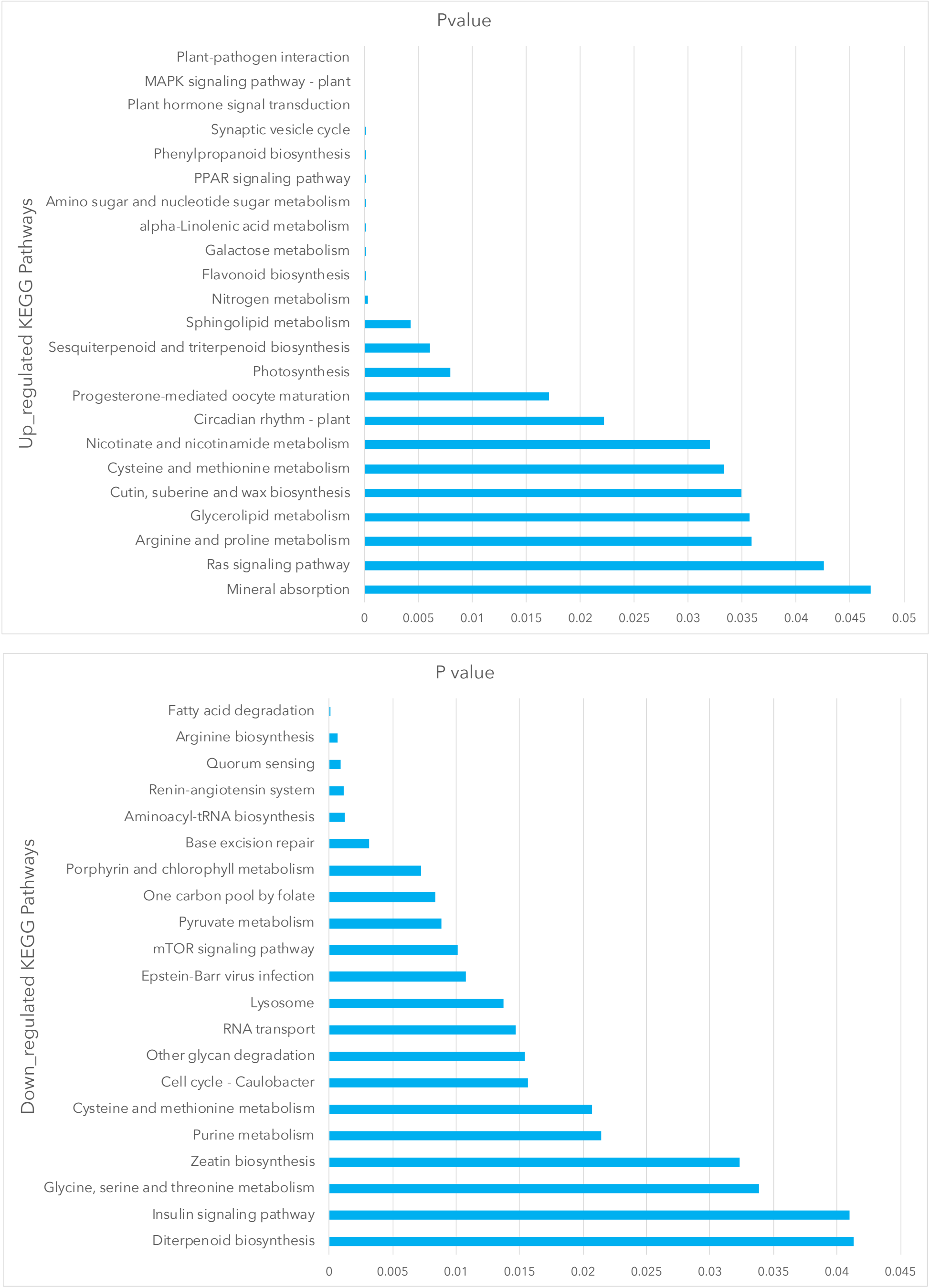

Figure S10. Kyoto Encyclopedia of Genes and Genomes (KEGG) analysis of upregulated (Top) and downregulated (bottom) transcripts in oceanside samples of *B. gymnorhiza*.
